# Increasing vaccine production using pulsed ultrasound waves

**DOI:** 10.1371/journal.pone.0187048

**Published:** 2017-11-27

**Authors:** Jida Xing, Shrishti Singh, Yupeng Zhao, Yan Duan, Huining Guo, Chenxia Hu, Allan Ma, Rajan George, James Z. Xing, Ankarao Kalluri, Isaac Macwan, Prabir Patra, Jie Chen

**Affiliations:** 1 Department of Electrical and Computer Engineering, University of Alberta, Edmonton, Canada; 2 Department of Biomedical Engineering, University of Bridgeport, Bridgeport, Connecticut, United States of America; 3 Department of Biomedical Engineering, University of Alberta, Edmonton, Canada; 4 Department of Electrical and Computer Engineering, University of British Columbia, Vancouver, Canada; 5 Department of Physiatry, University of Alberta, Edmonton, Canada; 6 School of Chinese Pharmaceutical Science, Guangzhou University of Chinese Medicine, Guangzhou, China; 7 Akshaya Bio Inc., Edmonton, Alberta, Canada; 8 Department of Laboratory Medicine & Pathology, University of Alberta, Edmonton, Canada; 9 Department of Mechanical Engineering, University of Bridgeport, Bridgeport, Connecticut, United States of America; Centre de Recherche en Cancerologie de Lyon, FRANCE

## Abstract

Vaccination is a safe and effective approach to prevent deadly diseases. To increase vaccine production, we propose that a mechanical stimulation can enhance protein production. In order to prove this hypothesis, Sf9 insect cells were used to evaluate the increase in the expression of a fusion protein from hepatitis B virus (HBV S1/S2). We discovered that the ultrasound stimulation at a frequency of 1.5 MHz, intensity of 60 mW/cm^2^, for a duration of 10 minutes per day increased HBV S1/S2 by 27%. We further derived a model for transport through a cell membrane under the effect of ultrasound waves, tested the key assumptions of the model through a molecular dynamics simulation package, NAMD (Nanoscale Molecular Dynamics program) and utilized CHARMM force field in a steered molecular dynamics environment. The results show that ultrasound waves can increase cell permeability, which, in turn, can enhance nutrient / waste exchange thus leading to enhanced vaccine production. This finding is very meaningful in either shortening vaccine production time, or increasing the yield of proteins for use as vaccines.

## Introduction

The hepatitis B virus (HBV) is the cause of the infectious Hepatitis B liver disease, which has caused epidemics in Asia and Africa [1]. The virus has infected about a third of the world population at one point in their lives [2]. Among them, 325 million are chronic carriers [3] and the carriers can suffer from acute hepatitis or chronic liver diseases like cirrhosis and even hepatomas (liver cancer) [4]. The HBV virus can spread from carriers to others. There are effective prophylactic vaccines on the market to protect individuals from HBV infection and control the spread of hepatitis B. At present, there are no therapeutic vaccines available on the market that can treat the chronic infection by inducing immune responses against HBV in chronically HBV-infected individuals. HBV S1/S2 antigens are good target antigens for developing a therapeutic vaccine to treat HBV carriers.

HBV vaccine was originally developed from HBV antigen (sAg) isolated from blood plasma of individuals who had long-standing Hepatitis B viral infection. The vaccines produced by this method have been used for about two decades, but the limitations are quite obvious, such as the high cost of production, limited availability of human plasma, poor acceptance rate and more importantly the risk of opportunistic infections [5, 6]. Current methods to increase vaccine production focus on synthetic recombinant DNA technology based on yeast expression systems, which are well established and widely used. However, serious adverse reactions, such as skin, rheumatic, vasculitic, hematologic, ophthalmologic and neurologic reactions, have been reported [7]. Using recombinant DNA technology based on baculovirus-insect cell expression systems (BCESs) is an alternative approach with several advantages:

1. It is highly versatile and can rapidly generate a wide range of complex and biologically active proteins for therapeutic vaccines [8]. The cultures are also easy to scale up because insect cells can grow in serum-free culture media without a CO_2_ incubator, which simplifies the purification process used to secrete proteins [9, 10, 11].
2. It is also considered safe for humans because insects are the host for baculovirus in nature and moreover, such viruses are non-pathogenic to humans [11]. Several insect-cell based proteins are currently used as therapeutic agents and vaccines (e.g., Provenge). The BCESs have been used to achieve high levels of expression of recombinant proteins not only for exploratory research, but also for commercial production. Currently, the insect cell based system is one of the major sources for recombinant protein production [9, 10].

Vaccines have become a highly effective approach to control contagious diseases in humans due to cost-efficiency and ease-to-implement [12]. However, due to the high cost associated with manufacturing these vaccines, existing vaccines are often not accessible in the developing world. Increasing vaccine production is an excellent approach to reduce the costs of the vaccines and promote extensive use, which will further help to control healthcare spending associated with infectious diseases and ease the financial burden worldwide.

**Fig 1:**
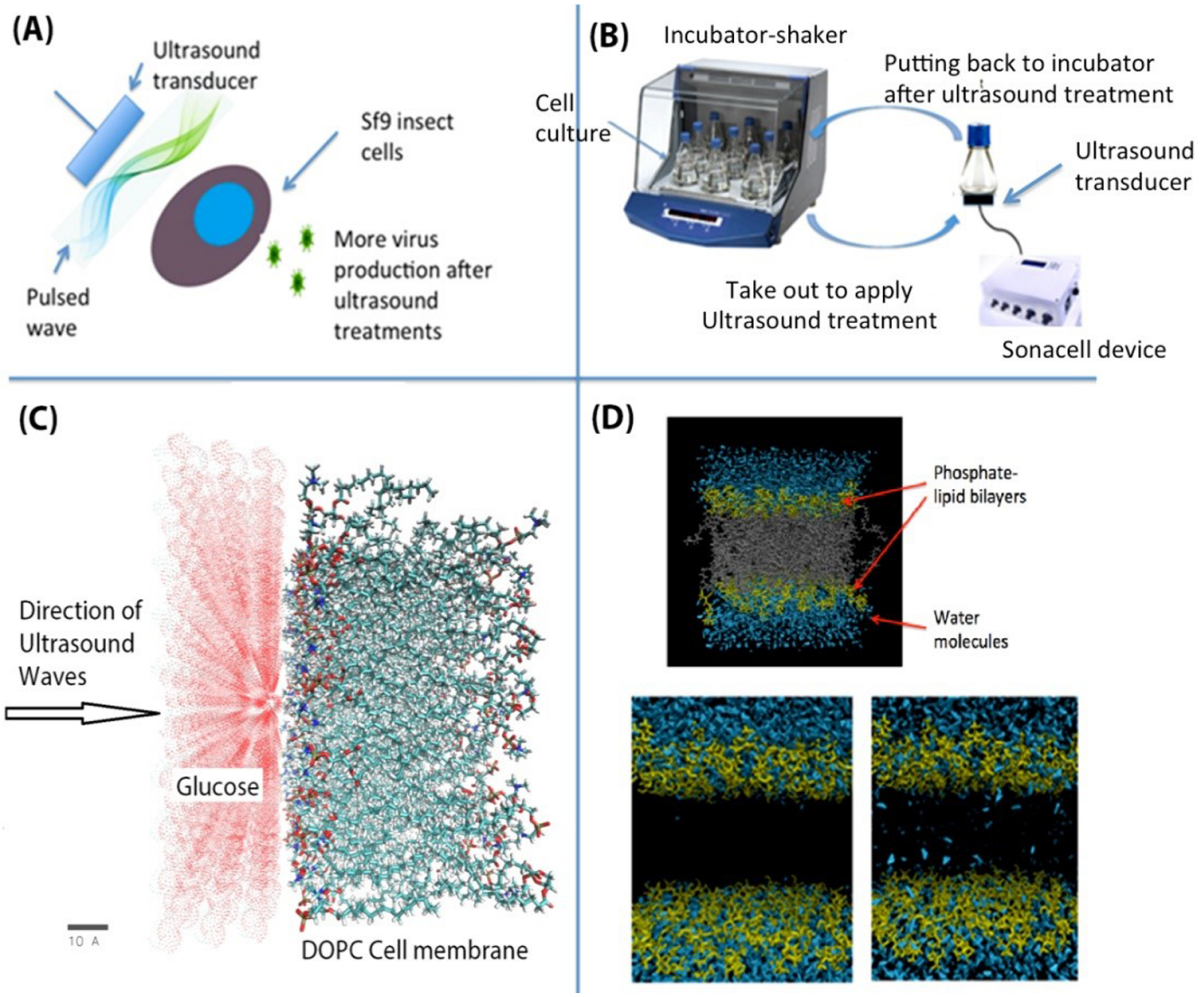
Increasing vaccine production using pulsed ultrasound waves: (A) Schematic of the proposed design; (B) Experimental setup to analyze the effect of ultrasound on the cell permeability; (C) Molecular dynamics model showing glucose molecules near the outer DOPC cell membrane; (D) A cell membrane/ water system built using CharmmGUI (before energy minimization). The blue colored balls are water molecules, yellow colored balls represent the head of phosphate-lipid bilayers and the grey colored balls represent the tail of lipids. During the permeation simulations, the hydrophobic tails are not shown for clarity.

Our lab has developed a low-intensity pulsed 1.5 MHz ultrasound (LIPUS) technology to increase vaccine production (Fig 1). 1.5 MHz was chosen on the basis of past successful outcomes across a variety of applications (listed in Table 1) and two US patents (8962290 B2 and 9012192 B2) were awarded. To the best of our knowledge, we are the first to publish on the use of sonication-based approach to increase vaccine production. Hepatitis B vaccines are used as a model system to demonstrate how LIPUS technology can be utilized to achieve this goal. Experimental results show that LIPUS induced ~27% increase in the expression of hepatitis S1/S2 surface (HBV S1/S2) antigen fragment fusion protein in Sf9 insect cells.

**Table 1:** 1.5 MHz ultrasound can help increase the growth of various biological species.

Investigations	Results
Why does 1.5 MHz pulsed ultrasound stimulate growth?	Using a common model microbiology system, *Saccharomyces cerevisiae*, we discovered that 1.5 MHz pulsed ultrasound can trigger metabolic effects beyond reprogramming of core pathway of carbon metabolism [13]
1.5 MHz pulsed ultrasound for algal lipid production	We achieved about 20% algal lipid increase compared to the control [14]
1.5 MHz pulsed ultrasound for CHO cell antibody production	We achieved about 30% antibody increase [15]
1.5 MHz pulsed ultrasound for bioethanol production	We achieved about 50% bioethanol increase [16]
1.5 MHz pulsed ultrasound for stem cell growth	(i) Enhanced the proliferation of fresh stem cells and maintained the viability of cryopreserved stem cells in vitro; (ii) did not affect the percentage of CD34^+^ and CD14^+^ cells; and (iii) enhanced burst-forming unit-erythroid colony formation [17]
1.5 MHz pulsed ultrasound for antibiotic production	We achieved about 90% Penicillium production increase [18]
1.5 MHz pulsed ultrasound for monoclonal antibody production	We achieved about 60% monoclonal antibody increase using hybridoma cells [19]
1.5 MHz pulsed ultrasound for dental tissue formation	The technology has passed the clinical trials and has been approved by Health Canada. The product is on market for orthodontic applications [20]

We also used NAMD to simulate the impact of impulse ultrasound waves on a cell membrane. Here NAMD refers to a widely used software package in computational chemistry and molecular modeling to perform calculations on both alchemical and free energy differences as well as steering the simulation across barriers [21]. More importantly, NAMD has become one of the best available software packages to simulate biomolecules such as lipids (Although cell membranes are composed of phospholipids and proteins, the most fundamental structure is phospholipid). Different from the simpler Monte Carlo simulations, MD is a more universal technique, especially in non-equilibrium ensemble systems and analysis of dynamic scenarios.

Despite the need for intense computing power, NAMD provides an efficient and quantitative way to investigate the impact of impulse ultrasound on cell membrane. Our simulation idea was initiated based on the mathematical model developed by Koshiyama *et al*. [22]. They predicted the velocities of water molecules affected by a shock wave. In our study, we constructed a regular lipid bilayer and assigned proper forces (calculated based on the proposed model) to the molecules less than 10 Å away from the cell membrane. Overall, the effect of pulsed ultrasound wave upon cell membranes was investigated at a molecular scale. The information gathered can help better understand the physical mechanism as to why LIPUS increases the protein expression. To validate our MD simulation results, a fluorescent glucose analogy, 2-NBDG (2-(N-(7-Nitrobenz-2-oxa-1, 3-diazol-4-yl)Amino)-2-Deoxyglucose), which is commonly used to monitor glucose uptake by cells was utilized [23,24]. It emits at 540 nm, which is visualized as a green light through optical filters of fluorescence microscope, when it was excited at 465 nm. We used fluorescence microscopy to compare the green color intensities of cells with and without LIPUS stimulations.

## Materials and methods

### Insect cells and media

Akshaya Bio Inc. (Edmonton, AB, Canada) kindly provided Sf9 insect cells, which were cultured and maintained in suspension in ESF 921 media (Expression Systems, Davis, CA95618, USA) at 27°C and 110 rpm. All shake flasks were purchased from Fisher Sci. (NH 03842 USA, Cat #: PBV125). In order to express the HBV S1/S2 protein, baculovirus encoding a 6xHis-tag HBV S1/S2 protein sequence was used to infect the insect cell at a Multiplicity of Infection (MOI) = 2, when the insect cell density reached 2 ~ 2.5 *10^6^ cells /mL. After 72 hours of infection the cell pellet was harvested by centrifugation.

### Ultrasound treatment

A LIPUS device (or a SonaCell device) designed in our lab was used to generate ultrasound at a frequency of 1.5 MHz and a pulse repetition rate of 1.0 kHz. The pulse duty cycle was 20% and the average intensity (the intensity used in this article refers to the intensity of spatial average temporal average, I_sata_) was adjustable up to 200 mW/cm^2^. In our experiment, we used a round transducer with a contact area of 3.5 cm^2^ (the diameter is 2.1 cm). Fig 1b shows the SonaCell device and the experimental setup. After the insect cells were inoculated into shake flasks, LIPUS was applied to stimulate the cells in the shake flask through the ultrasound transducer placed underneath each flask. Treatment dosages (ultrasound intensity, stimulation duration each treatment, and how many treatments per day) were adjusted according to the experimental setup. Culture flasks were placed back into the incubator-shaker after every treatment.

### Determination of cell numbers and protein production

#### Cell count

Viable and dead cells were counted using a hemacytometer with trypan blue staining. Both chambers of the hemacytometer from the same sample were counted and the average was used in this study. When we compared the control (without ultrasound treatment) and the ultrasound treated samples at each time point, we used the following equation:

$$\text{Increase}\;\%\;=\;\frac{\left(\text{Viable}\;\text{cell}\;\text{density}\;\text{with}\;\text{ultrasound}\;\text{treatment}-\text{Viable}\;\text{cell}\;\text{density}\;\text{in}\;\text{control}\;\text{sample}\right)}{\text{Viable}\;\text{cell}\;\text{density}\;\text{in}\;\text{control}\;\text{sample}}*100,$$ (1)

Eq (1) was used to calculate the increase of ultrasound treated sample over the control.

#### Protein analysis

Insect Sf9 cells were lysed in a lysis buffer (50 mM Tris-HCl, pH 7.5, 150 mM NaCl, 1 mM EDTA, 1% TritonX-100, and protease inhibitor cocktail (Cat no.: 539134, Calbiochem, Gibbstown, NJ, USA)). The lysate was then centrifuged at 12,000 xg for 15 minutes at 4°C and the supernatant was collected for use.

Samples of the protein were run on 12% SDS-PAGE homogenous gel and transferred to polyvinyl difluoride membrane (Bio-Rad Laboratories, Richmond, CA). The membrane was blocked in 5% milk at 4°C overnight, incubated for 1 h at room temperature with 1:2000 6xHis mAb with HRP Conjugate (Cat no: 631210, Clontech, Mountain View, CA 94043, USA). It was then visualized using an enhanced chemiluminescence assay (ECL kit, Amersham, London, UK).

To semi-quantitatively determine and compare the protein levels, blot images were analyzed using the ImageJ image analysis software (http://rsbweb.nih.gov/ij/index.html). Here, protein levels were quantified as the mean of integrated optical density.

### Ultrasound increases cell permeability

To confirm the hypothesis that LIPUS can increase cell permeability and thus increase nutrient uptake (for example glucose), we have designed the following experiment: 2-NBDG (Cat no: N13195, Thermo Fisher Scientific, Waltham, MA, USA) was used as a fluorescent probe to detect glucose uptake by cultured Sf9 cells. 0.1 mL of Sf9 cell culture at 5×10^6^ cells/mL were plated at two wells in a 12-well plate. The two groups of cell cultures were then diluted ten times with PBS to lower the concentration of glucose in the culture medium, and 0.5 mg 2-NBDG was then added to the two groups. After 15 minutes of incubation, one group of cells was stimulated with LIPUS at the intensity of 60 mW/cm^2^, while the other group was kept as a control without any ultrasound treatment. After another 15 minutes of incubation, two samples were centrifuged to recover cell pellets. The samples were then washed with PBS twice. Finally, the fluorescence microscope was used to measure the green colour intensity of the two groups of cells.

### Molecular dynamics model

For MD simulations, a solvent consisting of glucose was modeled using the CHARMM force field. The solution consists of 26 glucose molecules on one side of the cell membrane and the direction of the ultrasound waves in the form of the constant force on the glucose molecules as shown in Fig 1c (a model of a glucose/phospholipid system for glucose permeation through the cell membrane). There are 128 dioleoylphosphatidylcholine (DOPC) molecules in the membrane that was built using CHARMMGUI. Here, we did not model any glucose molecules on the other side of the membrane, or the inner membrane/cytoplasm of the cell.

#### Velocity of force modeling of the surrounding molecules

The purpose of doing the molecular dynamics simulation is to understand how ultrasound waves impact cell permeability. Based on the previous studies [19], better cell permeability results in better nutrient and waste exchange therefore healthier cells and cell growth. The initial idea was triggered by the simulation results published in [22,25], in which the authors simulated the interaction between the cell membrane and the shock wave. In our simulations, we assume that (i) cell membranes have thickness on the order of nanometers, and (ii) the velocities of the surrounding molecules of the buffer solution induced by the ultrasound waves are on the order of thousand meters per second. To accurately simulate molecular dynamics of such a system, a good estimation of the water molecule velocities and a constant force on glucose molecules by the ultrasound waves is required. Pulsed ultrasound wave was modeled as an impulse wave, with momentum per unit area *I* defined to be: $I\;=\;\int_{0}^{t}P(t)dt$, where *t* is the effective time of applying ultrasound, and *P* is the pressure close to the cell membrane. For example, if the intensity of the ultrasound is 300mW/cm^2^, the relationships between sound pressure *P* and sound intensity *I* can be defined using the following equations: $P\;=\;p_{0\;}10^{\frac{L_{p}}{20}}$ and $I\;=\;I_{0}10^{\frac{L_{p}}{10}}$, where *p*_*0*_ *= 2×10*^*−5*^ and *I*_*0*_ *= 10*^*−12*^, are the reference pressure and intensity, respectively. *L*_*p*_ is the sound pressure level in dB.

For simplicity, we assume that water molecules absorb all the momentums, and the ultrasound wave carries the velocity (*v)* of water molecules: $v\;=\;\frac{I\times A}{mN}$, where *m* is the mass of water molecule (3×10^-26^kg), *N* is the number of molecules in the simulation region, and *A* is the area of the cell membrane. For instance, if we have a cell membrane of area A≈40nm^2^, and 6400 water molecules, and the power of ultrasound applied is 300mW/cm^2^, we can easily calculate the ultrasound pressure *P* using the formula: $P\;=\;2\times 10^{-5}\times 10^{\frac{10\times log(\frac{I}{10^{-12}})}{20}}Pa\;=\;1095\;Pa$. Impulse *I* is estimated to be: I = 1095Pa×200μs = 0.219 Pa*s. Hence the velocities of water molecules are estimated to be $v\;=\;\frac{0.219*40*10^{-18}}{6400*3*10^{-26}}\;=\;4.56\times 10^{4}m/s$.

Using a similar approach for the selected ultrasound intensity of 60mW/cm^2^ for the glucose molecules (mass = 2.989*10^−22^ kg), the velocity of individual glucose molecule was found to be 0.888*10^4^ m/s. A constant force could be easily calculated using the equation: F = m*(v/t) to be 0.01315 pN. With a conversion factor of ~69.5pN = 1 kcal/mol/Å, the constant force turned out to be 0.19 cal/mol/Å that each glucose molecule will experience.

#### MD simulation setup

With a membrane surface area of ~40 nm^2^, 26 molecules of glucose (α–D–glucose) were appropriately positioned in the centre on one end of the phospholipid membrane. The crystal structure of glucose was extracted from a complex of a glucose binding protein taken from the protein data bank (PDB entry, 1N3Q). The glucose structure file was generated using CHARMM carbohydrate force field [26]. The 128 DOPC molecules (1,2-Dioleoyl-sn-glycero-3-phosphocholine, which is one of the most abundant lipid in animal cell) are organized to build the lipid bilayer system from the CharmmGUI (a powerful online cell membrane builder) membrane generator (*L*_*x*_ = *L*_*y*_, thickness of approx. 3.76nm). The entire DOPC/glucose system was dissolved in TIP3 water [27] in a water box (>47,000 water molecules). *Na*^+^ and *Cl*^-^ ions with a neutralizing concentration were added to the system to compensate for the net charge by ions. All starting structures initially underwent energy minimization using the steepest decent algorithm to remove the bad contacts and more importantly to reduce the overall system energy to the global minimum. After minimization, water molecules were removed from the system and the side of the DOPC membrane opposite to the glucose molecules was fixed (DOPC residues 65 to 128) to assure that the membrane itself would not be pushed by the constant force imparted by the glucose molecules. DOPC residues 1 to 64 were kept flexible to allow the glucose molecules to penetrate through the outer membrane. Atomistic steered molecular dynamics [28] was carried out in vacuum using the open-source software package NAMD version 2.10 with a time step of 0.05 fs. The CHARMM parameters for general force field of small molecules (CGenff) [29,30] were combined with the parameters for lipid [31] and carbohydrates [32,33]. A constant force of 0.19 cal/mol/Å was applied to all glucose molecules (steered molecular dynamics, SMD atoms). Periodic boundary conditions were applied in all three dimensions to avoid boundary effects caused by finite simulation system dimensions. Analysis was conducted using in-house routines and CHARMM analysis utilities. Molecular visualization was performed using VMD (Visual Molecular Dynamics).

The phospholipids consisted of 128 DOPC molecules surrounded by 6400 water molecules on both the sides. By performing energy minimization, we are able to minimize and relax the initially generated membrane structure. As we built our membrane using DOPC molecules, a production temperature of 300K was maintained. The purpose of applying heat is to mimic the increased speed of water molecules due to ultrasound treatments. MD production run was performed in conditions that temperature is controlled by using Langevin thermostat, while pressure is controlled by using the anisotropic Berendsen barostat, which are typical for simulating lipid bilayers in AMBER [34]. Each time constant counts as 0.02 ps as a leap-frog method was used to integrate the Newtonian equations of motion for water molecules. With water molecules assigned proper initial velocities by modifying the “.inpcrd” file, the periodic conditions were applied in *xyz* directions. Lennard-Jones interactions were cut off at a scale of 1.0 nm, while long-range electrostatic interactions were dealt with the particle mesh Ewald method with an Ewald coefficient of 0.27 [22]. The whole simulation was performed under the previously mentioned conditions for 5 ps with the SHAKE algorithm, which is the constraint algorithm in AMBER that solves linear constraint equations using Gauss-Seidel iterative method, in a simulation region with the size of 10 × 6 × 16 *nm*.

### Data analysis

Experimental values were determined in three to five replicas. All measured values were expressed as means and standard deviations (SD). The one-way analysis of variance (ANOVA) and Tukey multiple comparison post-test were used. Differences less than 0.05 (p<0.05) after correction were considered statistically significant.

## Results

### Selection of ultrasound treatment conditions

**Fig 2:**
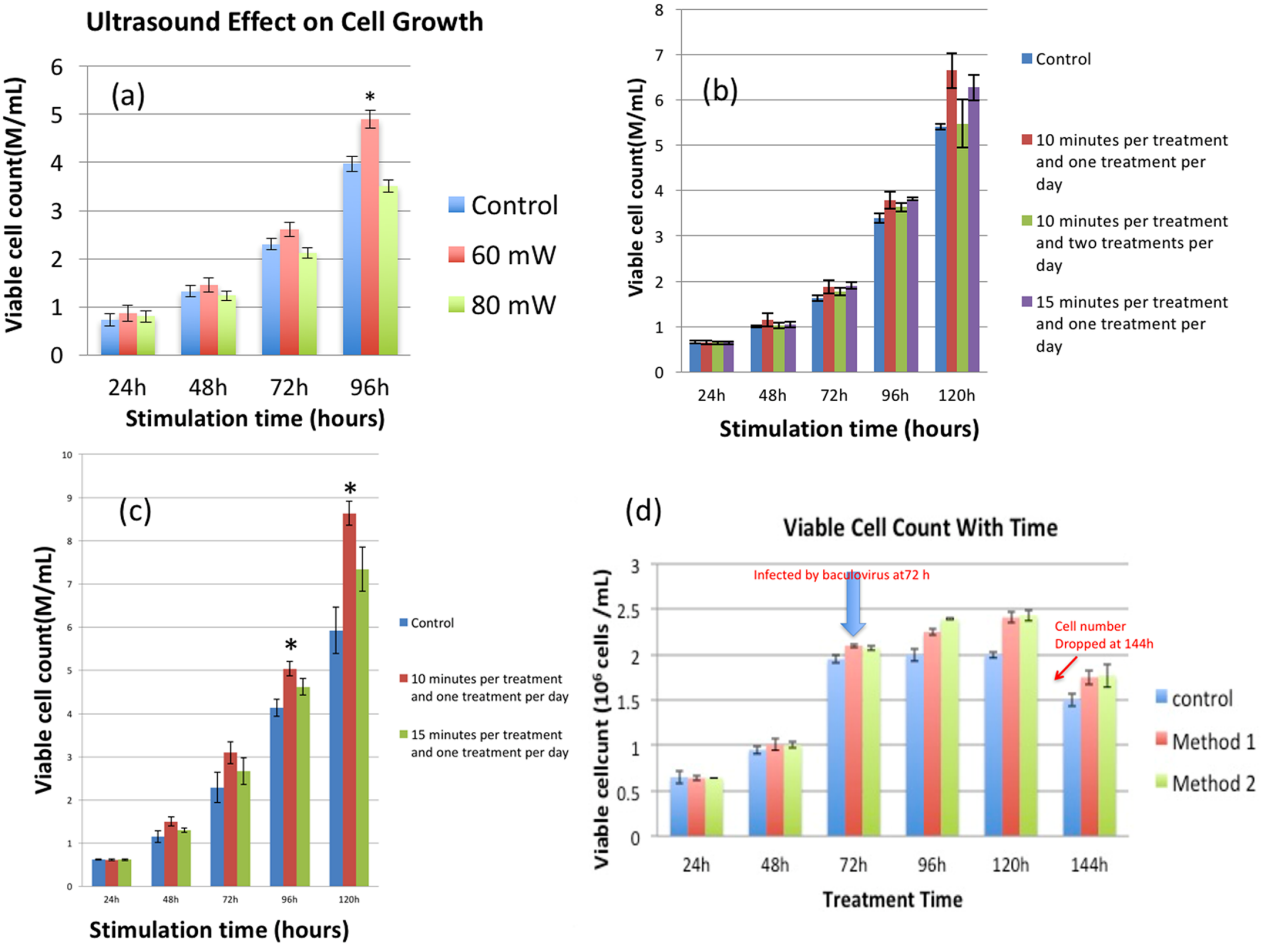
(a) We have changed the amplitude of ultrasound intensities and discovered that 60 mW/cm^2^ is better for Sf9 cell growth. (b) Insect cell growth in 30 mL media in a shake flask with various ultrasound treatments. (c) Insect cell growth in 30 mL media in a shake flask with 10 minutes and 15 minutes ultrasound treatments. For both (b) and (c), the control refers to insect cell culture without ultrasound treatment. The ultrasound intensity is 60 mW/cm^2^. (d) Cells’ growth curve. Method #1 involves stopping sonication after infection while method #2 is the one with continuous ultrasound stimulation. Cells were infected by baculovirus at 72 hours. Error bars highlighted with an asterisk mean there is a statistically significant difference between the ultrasound-treated sample and the control. *p < 0.05.

We have applied LIPUS to various cell systems such as hybridoma cells [19], stem cells [17] and CHO cells [15]. We discovered that the ultrasound intensity (I_sata_ = 60 or 80 mW/cm^2^) with 10-minute treatment was a better condition for cell growth. In this study, a quick screening experiment was performed to compare the treatment effects using 60 or 80 mW/cm^2^ on Sf9 insect cells and we discovered 60 mW/cm^2^ is better than 80 mW/cm^2^ (refer to Fig 2a). Therefore, 60 mW/cm^2^ was selected as ultrasound intensity for all the remaining experiments.

After we determined an appropriate ultrasound intensity, we then designed the following experiments to screen the suitable ultrasound treatment duration and treatment times per day. Although 10 minutes per treatment is good for the growth of most cell systems such as stem cells, Hybridoma cells, and CHO cells [17,18,19], we have tested 15 minutes to see whether prolonged stimulation can obtain better results.

Furthermore, we also tested treating twice a day to see whether more treatments per day can achieve better cell growth. We prepared four flasks to grow insect cells:

- One is the control (without ultrasound treatment),
- One is treated 10 minutes per treatment and once a day,
- One is treated 10 minutes per treatment but twice a day, and
- The last flask is treated 15 minutes per treatment and one treatment per day.

The viable cell density at each time point was counted and shown in Fig 2(b). After it was discovered that treating once per day is better than treating twice per day, we further designed another experiment to compare whether 10 minutes or 15 minutes per treatment is better. The results are shown in Fig 2(c). We found 10-minutes yielded better results. In all, we discovered that the longer treatment or multiple treatments per day does not translate to better yields (a possible cause might be cell fatigue).

### LIPUS affects protein expression

In this experiment, we used LIPUS to stimulate insect cells and then infected the cells with the recombinant baculovirus. Cells were harvested after 72 hours and then the protein level was determined by Western blotting. We designed the experiment with two ultrasound cultures to check ultrasound effect on protein production. We used LIPUS to stimulate cells to 2.0~2.5*10^6^ cells /mL. After infection, one culture sample did not receive ultrasound treatment anymore, while the other culture was still stimulated until harvest. Fig 2(d) shows the cells’ growth curve.

### Checking protein production increase

**Fig 3:**
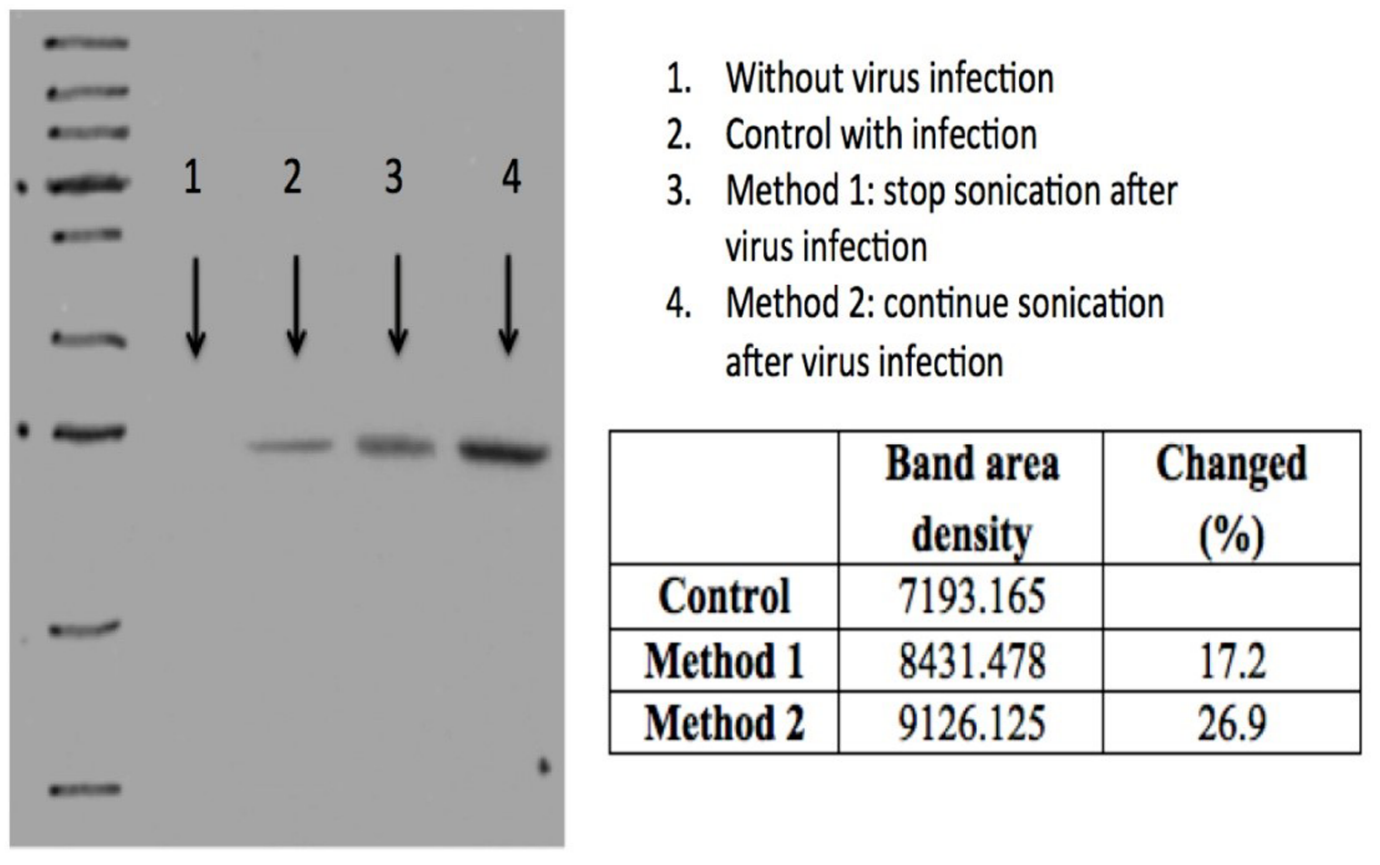
The Western blotting results. ImageJ software was used to measure the band area density change.

Fig 3 shows the Western blotting results. We used the software ImageJ to estimate band area density change. Results showed that protein production can be increased by 27% over the control after sonication.

### Cell permeability tests

**Fig 4:**
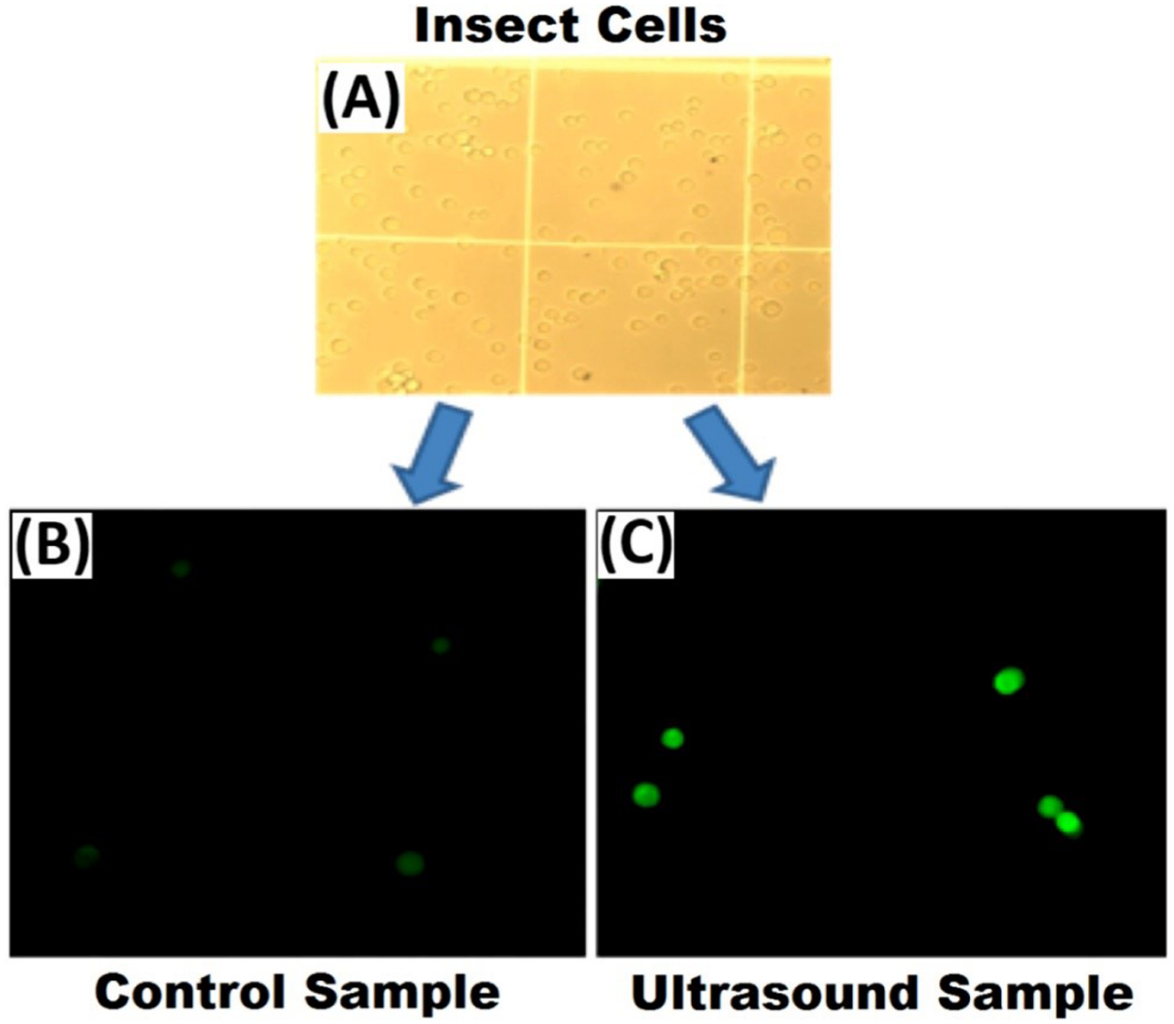
2-NBDG uptake in Sf9 cells with and without ultrasound treatment observed by conventional microscopy (A) and fluorescent microscopy (B and C). The sample with ultrasound treatment shows higher green intensity (C) compared to the control sample (A).

To explain the increase in vaccine production, we hypothesize that LIPUS can increase cell permeability by inducing transient openings in the cell membrane. In fact, it is quite difficult if not impossible to directly measure any cavitation of cells *in situ*. Therefore, we designed the glucose uptake experiment. The differences in the light intensity of cells in Fig 4(b) and 4(c) for the control and ultrasound treated samples indicate an increase in the uptake of 2-NBDG by the cells with the ultrasound treatment, which shows that ultrasound can indeed increase the glucose uptake in cells. Furthermore, our hypothesis is that ultrasound increases the glucose uptake by increasing the permeability of the cells. In addition, the increase of glucose uptake by the cells also provides the required energy needs for the cell growth and indirectly helps vaccine production. These results are in line with our expectations.

### MD simulation results of the penetration of glucose molecules

**Fig 5:**
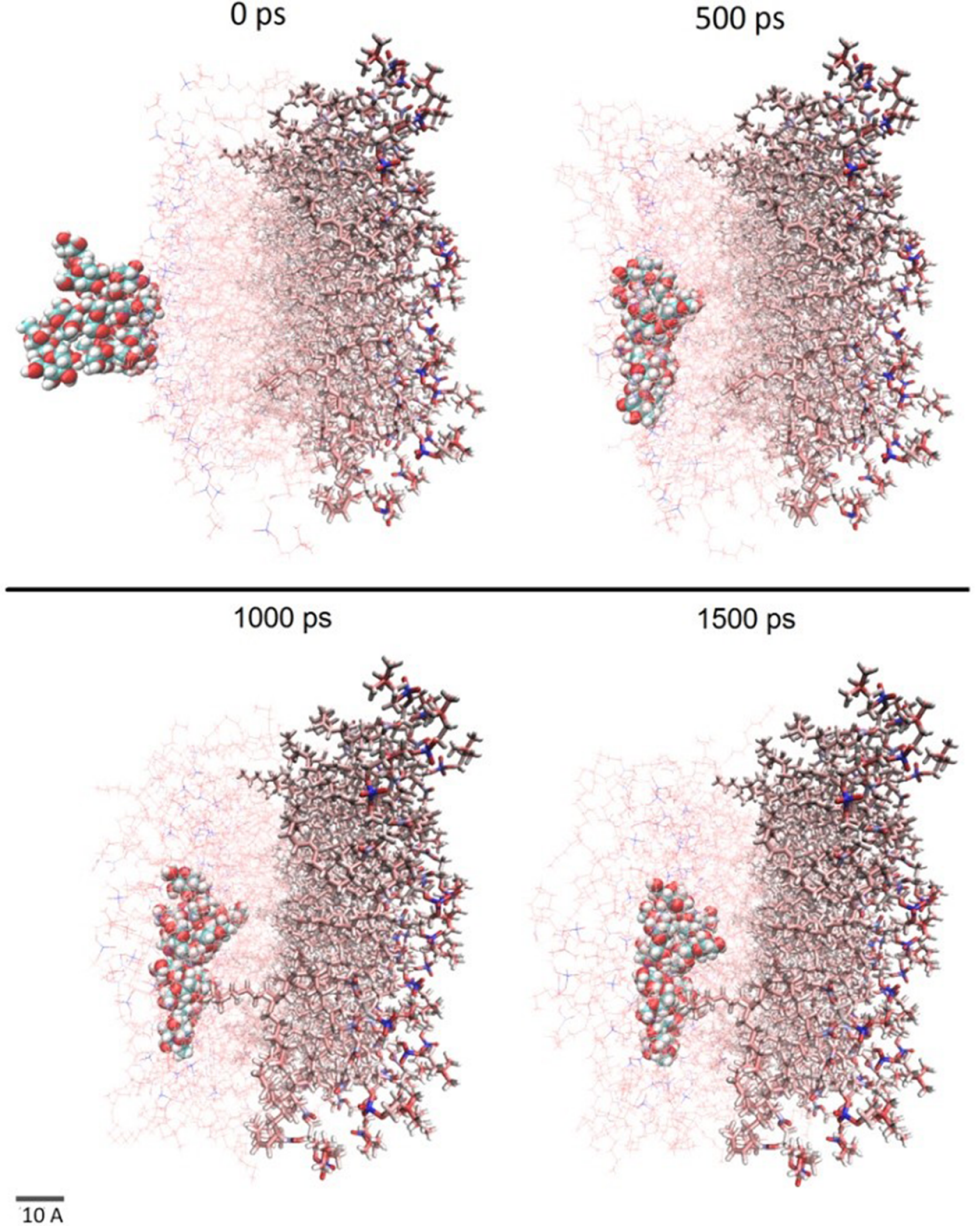
Representative screenshot showing the simulated trajectory of the glucose molecules through the DOPC membrane during the first 1500 ps.

Representative screenshots during the 1500 ps NAMD simulation run of glucose permeation through DOPC membrane is shown in Fig 5. It can be seen that over a period of 1500 ps, the glucose molecules pentrate through the outer 64 DOPC molecules (represented via lines to get a better view of the permeating glucose molecules) on their way towards the hydrophobic core. The membrane end towards the inner membrance (fixed DOPC residues 65–128) are shown using licorice representation.

**Fig 6:**
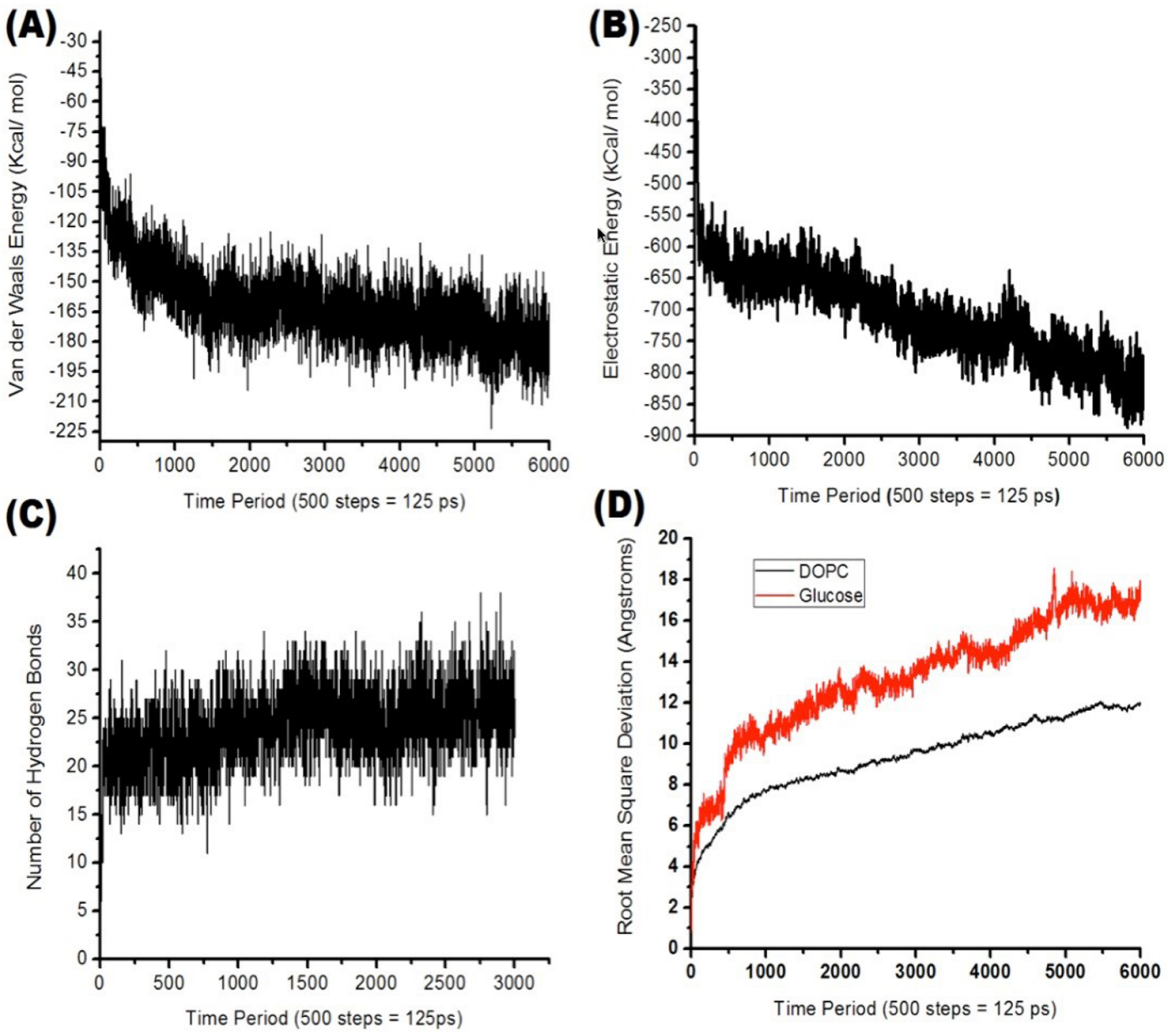
(A) Van der Waals energy between glucose and DOPC molecules; (B) Electrostatic energy between glucose and DOPC molecules; (C) Number of hydrogen bonds between glucose and DOPC molecules; (D) Root Mean Square Deviation for DOPC glucose molecules. All energies are based on the outer flexible DOPC residues (residue ID 1 to 64).

The non-binding interaction energies between glucose and DOPC molecules are plotted in Fig 6, which are based on Van der Waals energies, electrostatic interactions and hydrogen bonding. RMSD plots for both DOPC and glucose molecules are also presented to analyze the stability of glucose molecules as they penetrate through the cell membrane and also the energetically favorable rearrangement of the DOPC molecules that creates a channel for the incoming glucose molecules.

**Fig 7:**
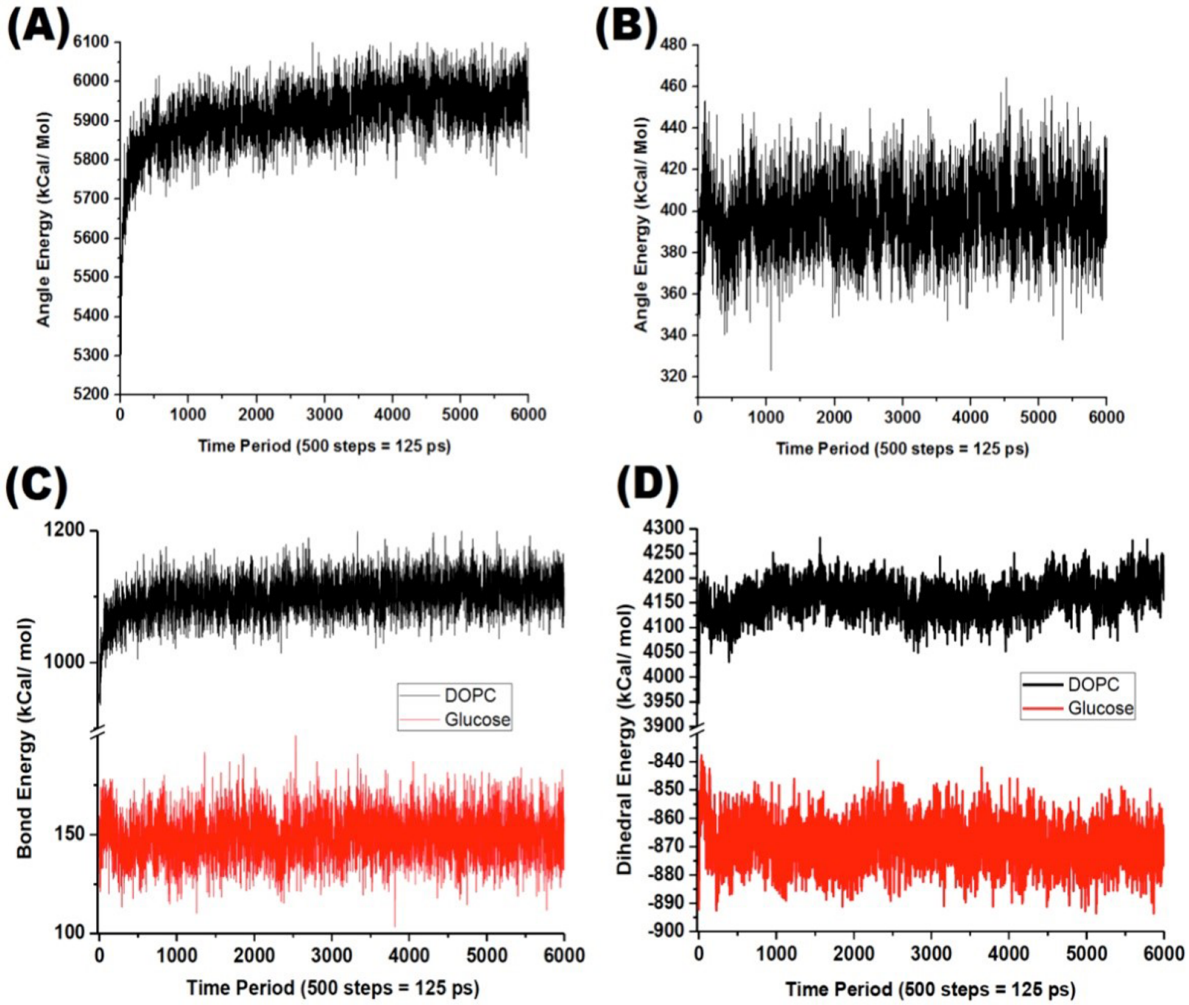
(A) Energy associated with the bond angles in the case of DOPC; (B) Energy associated with the bond angles in the case of glucose; (C) Bond energies for DOPC and glucose molecules; (D) Energy associated with the dihedrals in the case of DOPC and glucose molecules. All energies are for flexible DOPC molecules (residue ID 1 to 64).

Similarly, the energies associated with the bonds, angles and dihedrals for both the DOPC and glucose molecules are also plotted as shown in Fig 7. Such energies are mainly used to analyze the conformational changes in the DOPC molecules and also the stability of the glucose molecules during the application of ultrasound waves in the form of constant force vectors.

## Discussions

### Higher ultrasound intensity resulting in lower cell growth

Based on our previous studies over the years (refer to previously listed references [13–20]), it is important to choose a right dosage of ultrasound stimulation to achieve the optimal results. If the intensity is too high, the ultrasound can have an adverse effect on cell growth, even damaging the cells. The dosage includes parameters such as intensity, duration of ultrasound treatment and how many times of treatments per day. Different cell types respond to the ultrasound treatment differently. Higher ultrasound intensity can even kill cells. In Sf9 insect cells, 60 mW/cm^2^ happens to be better in helping cell growth than 80 mW/cm^2^.

### One treatment per day is better than two treatments per day

From Fig 2(b), we can see that the maximum increase in cell density over control is 23% at 120 hours for the group with 10-minute treatment per day and 16% for the group with 15-minute treatment per day. There is no significant increase, however, for the group with 10 minutes per treatment and two treatments per day. Overall, one treatment per day shows better results than two treatments per day for the growth of insect cells. One of the possible reasons could be that the cell membrane of Sf9 is delicate. Although the proper ultrasound stimulation increases the cell permeability, which promotes the cell metabolism and leads to the cell number increase, the Sf9 cell membrane still needs some time to recover after the ultrasound treatment. Two treatments per day seems too much that can break the cell membrane and affect further increase in the cell count.

### Treatment of 10 minutes per day is better than that of 15 minutes per day

Results in Fig 2(b) and 2(c) also confirm our observation (“One Treatment Per Day Is Better Than Two Treatments Per Day”) obtained from the previous experiments (refer to Fig 2(a)). From the experimental results in Fig 2(a) and 2(b), we can conclude that 10-minute treatment per day is better than 15-minute treatment per day for the growth of insect cells. We also found that applying ultrasound at an early stage of cell growth is not good for the multiplication of insect cells. Therefore, in all our experiments, we let the cells grow for 24 hours after inoculation and then applied the ultrasound stimulation.

### Is continuous ultrasound stimulation better than no stimulation after infection?

Fig 2(d) shows that the infection by baculovirus affects insect cell growth. After adding the virus at 72 hours, the cell number in all the groups begins to increase slowly. At harvest (144 hours) or after 72 hours from adding the virus, we discovered that the cell number in all the groups drop dramatically since a number of cells died due to the virus infection. Fig 2(d) also shows that method 2 (or continuous LIPUS stimulation after infection) is better than method 1 (or no stimulation after infection).

### Protein production increase

We define the Cell Productivity (CP) as the production of protein per cell, and we assume the blot band density is the concentration of protein produced from the insect cell. We can calculate the insect cell's CP by the following equation:

$$CP\;=\;\frac{Band\;density}{Viable\;cell\;density\;at\;the\;time\;of\;infection}$$ (2)

We can compare the CP of insect cells with and without ultrasound treatment, and the calculated CP values are listed in Table 2. Ultrasound stimulation can increase the cell's productivity.

**Table 2:** Cell productivity increase after sonication.

	Infection density (cells/mL)	Band area density	CP	Increase over control (%)
**Control**	2.130*10^6^	7193.165	0.034	
**Method 1**	2.345*10^6^	8431.478	0.036	6.47
**Method 2**	2.345*10^6^	9126.125	0.039	15.24

When cells were infected, we either continuously applied ultrasound (method 2) or stopped sonication (method 1) (refer to Fig 2(d)). We did not observe significant differences in cell number between one with ultrasound stimulation until harvest vs. the one with continuous sonication (only at 96 hours, the cell number using method 2 was statistically better than that using method 1). However, from the Western blotting results (refer to Fig 3), we indeed discovered that continuous ultrasound stimulation increased protein production by 15% (method 2) while stopping sonication after infection (method 1) increased the productivity by almost 6%.

### Ultrasound increases cell permeability to glucose molecules

Fig 5 shows the 1.5ns SMD simulation run visually indicating how glucose molecules would penetrate through the cell membrane. Analysis of the non-binding energies from Fig 6 show Van der Waals energies (Fig 6a), electrostatic interactions (Fig 6b) and the making & breaking of hydrogen bonds (Fig 6c) as crucial factors facilitating the penetration of the glucose molecules through the cell membrane. It can be seen that the attractive Van der Waals and electrostatic interactions would move the glucose molecules forward as successive inner residues in the cell membrane attract the glucose molecules with stronger energies. As glucose molecules move through the cell membrane they also temporarily bind with the DOPC molecules via hydrogen bonds (Fig 6c), keeping the molecules energetically stable. From Fig 6(d), it is confirmed that glucose molecules move ~17 Å (halfway through the cell membrane) under the effect of ultrasound pressure. In contrast to this, DOPC molecules have ~10 Å of RMSD indicating that cell membrane is energetically stable and would not fall apart due to the forced passing of glucose molecules.

Similarly, analyzing the plots in Fig 7(a) and 7(b), it is found that there is a relatively larger variation in the bond angle energies for DOPC molecules (~200 kcal/mol) compared to glucose molecules (~ 70 kcal/mol). This indicates that the cell membrane slowly makes way for the permeating glucose molecules to move forward towards the hydrophobic core and eventually towards the inner membrane. Similarly, larger variations in the bond energies for glucose compared to DOPC (Fig 7c) indicate the role of temporary bonds that drive the glucose forward through the membrane. Furthermore, it is important to notice that both bond energies and bond angle energies are repulsive (positive) in nature indicating that only non-binding hydrogen bonds are primarily used by the glucose molecules to pass through the cell membrane. Finally, it is also seen from Fig 7(d) that dihedrals in the case of DOPC molecules have a larger variations in energies (~ 140 kcal/mol) compared to glucose (~ 40 kcal/mol), and this can be attributed to the DOPC molecules slowly moving out of way from the path of incoming glucose molecules.

## Conclusions

LIPUS offers a promising way to stimulate HBV S1/S2 production and can achieve about a 27% increase. Continuous sonication is observed to perform better than stopping LIPUS stimulation after infection. Theoretically, we could exhaustively try all combinations (intensity, duration of ultrasound treatment, and the number of treatments per day) to find the optimal growth condition. However, it is not the purpose of this article. Our innovation is to show that the LIPUS technology can help increase vaccine production. Overall, we postulate: LIPUS increases cell permeability due to cavitation, which causes glucose to enter the cell. However, it is known that the mechanism for glucose uptake into the cell is facilitated diffusion which involves proteins such as GLUT4. Typically glucose binds with GLUT4 as it penetrates through the cell membrane that makes the transportation of glucose smoother. Therefore, LIPUS can also be seen as possibly increasing the rate at which glucose binds GLUT4 thereby increasing the concentration of glucose within the cell. All our simulation results do support that hydrogen bonds are formed between glucose and the membrane. Due to the formation of these hydrogen bonds, glucose molecules should get stuck within the membrane, as the bonds hold them there. Nevertheless, this does not happen because of the proteins causing facilitated diffusion, whose production rate is increased by LIPUS. This method can either reduce vaccine production time or increase vaccine productivity.

## Supporting information

S1 File Raw data for Fig 2a. (XLSX)

S2 File Raw data for Fig 2b and 2c. (XLSX)
